# Real-world experience with family beliefs and organizational and communication management in Colombian parents of children with leukemia: a qualitative study

**DOI:** 10.3389/fpsyg.2026.1783044

**Published:** 2026-04-01

**Authors:** Luz Helena Buitrago-León, Yolanda Pastor, Domingo Palacios-Ceña

**Affiliations:** 1 International Doctoral School, Rey Juan Carlos University, Móstoles, Spain; 2 Research Group in Health, Sport and Clinical Psychology, Faculty of Psychology, Universidad El Bosque, Bogotá, Colombia; 3 Department of Psychology, Research Group on Health and Psychosocial Well-Being in Adolescence and Youth (WELL-YOUTH), Universidad Rey Juan Carlos, Alcorcón, Spain; 4 Department of Physical Therapy, Occupational Therapy, Physical Medicine and Rehabilitation, Research Group of Humanities and Qualitative Research in Health Science (Hum&QRinHS), Universidad Rey Juan Carlos, Alcorcón, Spain

**Keywords:** childhood cancer, family beliefs, family communication, family resilience, leukemia, organizational processes

## Abstract

**Background/objectives:**

Coping with childhood cancer presents a major challenge for families. Some studies suggest that this experience strengthens families, leading to resilient processes, as observed by Froma Walsh. This study describes family resilience strategies for coping with childhood leukemia during the first year after diagnosis in the Colombian context, applying the model designed by Walsh.

**Methods:**

We conducted a qualitative study following a constructivist paradigm. The sample consisted of 10 Colombian parents with a child between eight and 12 years of age who were pediatric oncology patients diagnosed with leukemia. We used an intentional (non-probabilistic) sampling through a non-profit organization in the city of Bogota. In-depth interviews were conducted. The study was stopped when data saturation was reached. We then applied a thematic and conversational analysis.

**Results:**

Our data show that resilience processes coexist with negative narratives. Although a childhood cancer diagnosis is a negative experience for families, many parents manage to reinterpret the situation in a positive light and face the experience with optimism. In the Colombian context, religious beliefs help give meaning to this experience. We identified more difficulties related to the organizational processes, due to inconsistencies in the healthcare system in their management of this problem, in addition to a strong influence of cultural beliefs regarding parental roles.

**Conclusion:**

The results obtained provide a comprehensive overview of the factors that favor the construction of adversities within the family nucleus of a child with leukemia. These findings can be used to design intervention strategies to promote resilient experiences.

## 1 Introduction

Facing a diagnosis like cancer is not an easy task for any family. A diagnosis in a child or adolescent can be even more difficult in a Western society (Lewandowska, 2022), where cancer is still synonymous with death and is an unexpected process at the beginning of one’s life (Galicia Pacheco et al., 2024). In the case of childhood physical illnesses, the impact and dependence on the family system are even more intense (Moscato et al., 2024). The role of parents in pediatric care is fundamental, as the child’s health and wellbeing are linked to physical, emotional, and social healthcare circumstances, as well as to parenting practices (Bradshaw et al., 2019; Hinton et al., 2022). A recent review highlighted the importance of family functioning as a risk or protective factor for the quality of life and wellbeing of children with cancer (Neugebauer and Mastergeorge, 2021). In this sense, parental stress negatively influences children’s cognitive functioning over time (Willard et al., 2016), their quality of life (Loiselle et al., 2016), and their emotional and behavioral adjustment (Colletti et al., 2008). Poor family functioning is also negatively associated with a poorer quality of life (Zheng et al., 2018) and psychosocial adjustment (Jobe-Shields et al., 2009) in children with pediatric cancer. Conversely, greater family cohesion is associated with less distress in both children with cancer and their siblings (Jobe-Shields et al., 2009; Prchal and Landolt, 2012).

A theoretical model that offers a positive view of family functioning in the face of adversity has been proposed by Walsh (2002). This author argues that, although some families in these situations can be torn apart by crises or chronic tensions, many others emerge stronger and with more resources. In Walsh’s model, resilience is understood as “the ability to withstand and rebound from disruptive life challenges […] It involves dynamic processes fostering positive adaptation within the context of significant adversity” (Walsh, 2002, p. 130). This ability of families to adapt to adversity and emerge stronger is termed “family resilience.” Walsh’s model identifies three key processes that help families adapt to adverse situations: (1) the belief system, i.e., how the family interprets and makes sense of difficult experiences; (2) organizational patterns, i.e., how the family structures and reorganizes itself in the face of stress; and (3) communication processes that encourage the expression of emotions, coordination of actions, and maintenance of family cohesion. Walsh’s model (2002) has led to a reinterpretation of the process of coping with adverse situations in the family. This rethinking repositions the family from a more hopeful perspective, projecting the possibility of growth, as opposed to a second view that focuses on suffering and stress and highlights the importance of identifying and measuring risk and protective factors to strengthen the resilience of the entire family system throughout the course of the illness (Walsh, 2002).

Walsh’s model has been widely applied to pediatric oncology contexts. Building on this framework, for example, various researchers found that most families of pediatric cancer patients develop resilience, although the extent of this resilience is shaped by several clinical and relational factors (Navea Martín and Tamayo Hernández, 2018; Rueda and Cerezo, 2020). Specifically, resilience tends to be lower when the diagnosis is recent, when the tumor type or prognosis is more severe, and when communication between families and healthcare professionals is limited. Among the relational processes identified in Walsh’s model, family communication emerged as one of the strongest predictors of resilient adaptation, underscoring its central role in helping families reorganize, make meaning of the illness, and engage in collaborative coping.

Extending this evidence base, Marbot et al. (2025) confirmed that both parents and children with cancer show medium-to-high levels of resilience, with social support emerging as one of the most consistently protective factors. Stress and other individual vulnerabilities, in contrast, appear to function as risk factors. Similarly, in their systematic review, Qiu et al. (2024) reinforced the relevance of the three domains of family resilience proposed in Walsh’s model when a family member faces cancer. They found that a sense of coherence, a positive appraisal of the illness, an acceptance of uncertainty, spirituality, and religious beliefs were positively associated with family resilience. Regarding organizational processes, family commitment and social support had the strongest empirical evidence, while harmonious communication also emerged as a key protective factor for resilient family functioning.

Although research on family resilience in the context of childhood cancer has expanded in recent years, the existing evidence remains overwhelmingly quantitative, which limits the field’s ability to capture the subjective, evolving, and context-dependent experiences of families during the first year after diagnosis (Marbot et al., 2025; Navea Martín and Tamayo Hernández, 2018; Qiu et al., 2024). Qualitative studies are still scarce, and the few that exist have been conducted primarily in non-Latin American settings. For example, a longitudinal qualitative study done with families of children with leukemia in China identified a continuous and complex adaptation trajectory that unfolds across three stages in the first year after diagnosis (Huang et al., 2025). Other studies using mixed methodologies showed that North American families who have gone through this process demonstrate a greater mastery of coping and resilience strategies in families whose children survive (Rosenberg et al., 2014), while in Turkey, parents reported experiencing significant emotional stress and major changes in family roles, finding it difficult to pay attention to siblings. Protective factors in these circumstances include robust support networks, empathetic healthcare professionals, and adaptive coping strategies (Güney Yılmaz et al., 2025). However, research in Latin America remains limited and fragmented, despite evidence suggesting culturally specific dynamics. For instance, a Colombian study based on the life stories of mothers reported that resilience develops throughout the illness, but becomes strained during the periods of greatest suffering for the child (Genes Núñez et al., 2025), whereas a longitudinal qualitative study by González Salazar (2019) emphasized processes that resignify family dynamics, including the adoption of new roles, strengthened bonding, and improved communication. Taken together, these gaps in the research reveal a lack of qualitative and context-sensitive studies in Latin America that examine how families navigate the early stages of childhood cancer within their sociocultural environment. To address this gap directly, the present study describes the resilience strategies used by Colombian families to cope with childhood leukemia during the first year after diagnosis, drawing on Walsh’s family resilience model (2002) to identify culturally grounded, context-specific processes.

## 2 Methods

### 2.1 Design

We conducted a qualitative study based on a constructivist paradigm (Creswell and Poth, 2018; Ritter et al., 2023), which describes how people construct their own social reality and knowledge through reconstructions of their individual experiences (Creswell and Poth, 2018). In a constructivist paradigm, knowledge is understood as socially constructed, negotiated, and shaped through interactions between individuals and their environments (Dahal, 2025). This ontological and epistemological stance influences how data are obtained and interpreted, as well as the role of researchers throughout the research process. From this perspective, interview data are not treated as neutral or objective representations of reality. Instead, they are viewed as co-constructed narratives arising from the dynamic interaction between the participant and the researcher (Dahal, 2025). The meanings expressed by participants are considered context-dependent and influenced by their personal histories, values, and social positions. Likewise, the researcher’s perspectives, prior assumptions, and interpretive frameworks actively shape how those meanings are elicited, recognized, and analyzed. Consequently, the interpretive process becomes reflexive (Dahal, 2025; Olmos-Vega et al., 2023). The researcher acknowledges that their presence, questions, and interpretive decisions form part of the knowledge production process. The goal is to understand how participants construct their experiences and make sense of the phenomena under study. For this reason, in this qualitative study, in-depth interviews were used as data collection instruments to gather participants’ individual experiences and perspectives. The context of healthcare delivery for children with cancer in Colombia was taken into consideration, and strategies were incorporated to document the researchers’ reflexive process, including researcher positionality and the use of reflexive notes (Olmos-Vega et al., 2023). The study followed both the Standards for Reporting Qualitative Research (O’Brien et al., 2014) and the Journal Article Reporting Standards for Qualitative Research (Levitt et al., 2018).

### 2.2 Research team and reflexivity

Before conducting the study, the researchers defined their position by outlining the theoretical framework, their guiding beliefs, the motivation behind the study, and key details about the research team (See Table 1). Researcher positionality is an integral component of the reflexive process, as it makes explicit the research team’s starting point and thereby enables an assessment of how they may influence – or be influenced by – the context and the participants (Creswell and Poth, 2018; Dahal, 2025). In qualitative inquiry, researchers are not neutral instruments in data collection or analysis. Rather, they participate actively in the construction and interpretation of meaning throughout the research process (Levitt et al., 2018).

**Table 1:** Team reflexivity.

Topics	Description
Theoretical framework	In qualitative research, the constructivist paradigm posits that reality is socially constructed, emphasizing the subjective meanings that individuals assign to their experiences. Researchers engage in interpretive inquiry, acknowledging multiple realities shaped by context, interaction, and perspective. This approach values participant voices and seeks to understand phenomena through co-constructed narratives.
Beliefs	Influenced by Walsh’s model (2002), the general belief guiding this study is that families find and develop coping resources in the process of a child’s cancer, despite the stress and suffering the family may be experiencing. Changes in the family belief system, organizational patterns, and communication processes are expected to be found. Religion and spirituality are also expected to play an important role in the Colombian context.
Motivation behind the study	The main motivation is to gain a better understanding of the families going through these processes in order to design interventions that promote and improve family resilience.
Research team	The team is made up of three researchers (two women), two psychologists, and a researcher-nurse. All the researchers had experience in the development of qualitative studies. None works in a clinical setting.

### 2.3 Context

The context is described in Supplementary material (section 1).

### 2.4 Participants

The study sample consisted of 10 Colombian parents with a child between eight and 12 years old who is a pediatric oncology patient. The type of sampling was intentional, considering inclusion criteria such as the child’s age range, a diagnosis of leukemia within one year at the time of the interview, and the subjects’ informed consent. The location of the sample was decided with the non-profit Colombian Foundation for Leukemia and Lymphoma, located in the city of Bogota, during 2021–22. Participants ceased to be included once data saturation was reached (Sebele-Mpofu, 2020). Data saturation refers to the point in the data collection and analysis process at which incoming data contribute little to no new information, participants begin to repeat what has already been expressed, and/or no new themes emerge from the dataset (Sebele-Mpofu, 2020). The saturation process was characterized by conducting data collection (interviews) and analysis before proceeding with the next interview, following an iterative approach. This procedure made it possible to compare analytical findings across successive interviews, allowing the researchers to determine whether the content was new or repeated (Dahal, 2025). Previous qualitative studies (Hennink and Kaiser, 2022; Wutich et al., 2024) indicate that data saturation is typically reached between nine and thirteen interviews. In the present study, saturation occurred with participant number ten.

Ten parents of children with leukemia were recruited (five females, 50%). The mean age was 37. Table 2 presents the sociodemographic data of the sample.

**Table 2:** Sociodemographic data of participants.

Category	Male (*n* = 5)	%	Female (*n* = 5)	%
Age				
18–30	1	20%	3	60%
30–40	2	40%	2	40%
40–50	1	20%		
>50	1	20%		
Marital status				
Single			2	40%
Married	3	60%	1	20%
Separated			1	20%
Domestic partnership	2	40%	1	20%
Education level				
High School			2	40%
Technical	4	80%	1	20%
Undergraduate			2	40%
Doctorate	1	20%		
Socioeconomic level*				
One			3	60%
Two	1	20%		
Three	3	60%	1	20%
Four	1	20%	1	20%
Occupation				
Self-employed	3	60%		
Employed	1	20%	1	20%
Homemaker			4	80%
None	1	20%		
Family structure				
Nuclear	5	100%	4	80%
Non-nuclear			1	20%
Health system				
Contributory	5	100%	3	60%
Subsidized			2	40%
Household economic provider				
Father	3	60%	3	60%
Mother	1	20%		
Both	1	20%	1	20%
Other			1	20%

*In Colombia, the National Planning Department classifies the population in six socioeconomic strata: 1. Low-Low; 2. Low; 3. Medium-Low; 4. Medium; 5. Medium-High; 6. High. **Colombia has two health insurance systems: contributory (which requires an employment contract) and subsidized (people considered poor and vulnerable).

### 2.5 Ethics statement

This study was reviewed and approved by the ethics committees of the two institutions involved. Eligible parents were notified and their informed consent obtained. Those who chose not to participate were excluded from the sample, and the sample was established with only those who voluntarily signed the informed consent form.

### 2.6 Data collection

We conducted semi-structured interviews based on Walsh’s (2004) proposal. The guide was validated by expert opinions. The procedure consisted of the selection of three experts in oncology and psychometric aspects using the focus group method (as described in Bryant, 2025). Subsequently, the experts validated the content of the instrument based on the categories of sufficiency, clarity, coherence, and relevance, which led to an analysis of agreement between the judges and adjustments to the interviews in terms of the semantics of the questions and a reduction in the number of questions when they were repeated. The pilot project took place one month prior to the final application.

Supplementary material (section 2) contains the question guide based on Walsh’s (2004) framework. The interviews were conducted in Spanish, with audio recordings as additional support for the transcriptions, prepared by a trained transcriber and verified for accuracy. As an investigative element, the transcripts were anonymized by changing the names to alphanumeric codes.

### 2.7 Analysis

Regarding the data analysis, this article applies a qualitative narrative study. We conducted a thematic deductive analysis following the processes and categories of Walsh’s (2004, 2016) model described in Table 3. The theoretical categories connect to three important categories for the study of family resilience: the family belief system, organizational patterns, and communication processes. Additionally, we used these categories in an initial analysis to classify the narrative content analyzed.

**Table 3:** Processes and categories of the Walsh’s model (2002).

Processes	Categories
Belief systems	Making meaning of adversity
	Positive outlook
	Transcendence and spirituality
Organization patterns	Connectedness
	Flexibility
	Social and economic resources
Communication processes	Open emotional sharing
	Clarity
	Collaborative problem solving

The coding process consisted of several stages. First, the transcribed interviews were read and re-read. In the second stage, the fragments of the text containing information relevant to the research question/objectives were selected. In the third stage, codes or descriptive units were identified within the selected textual fragments. Each text was coded using the open, axial, and selective coding strategy (Strauss and Corbin, 1990) and categorized by identifying one or more text narratives with a theme and relating it to a code (short for a theme idea) for each (Gibbs, 2007). The fourth stage consisted of the deductive grouping and organization of the codes based on the key processes of Walsh’s (2016) model. Fifth, once the codes had been organized according to the model, the new content was inductively identified. Then, the research team analyzed the thematic content, using an inductive method to identify, organize, and thoroughly analyze the themes by reading and re-reading the information (Braun and Clarke, 2006). In this way, although the codes were grouped according to a pre-existing theoretical model, this approach allowed for the inductive construction of categories and the identification of new content that had not been established previously. See Supplementary File 3 for the conceptual mapping. As part of the analysis process, the research team held a series of meetings. These sessions involved collectively examining the organization of the text fragments that contained relevant information, discussing the rationale for the categorization and identification of codes, and evaluating the consistency of the coding decisions between the various researchers. In addition, the team collaboratively explored the emergence of new content and themes, reflecting on how these inductively generated insights complemented or extended the initial coding framework. These sessions also served to enhance reflexivity, enabling researchers to critically appraise their interpretive assumptions and ensure that the analytical process remained rigorous and transparent. When differences were found, gradual argumentative analyses were performed until the researchers reached a consensus (Alhojailan, 2012).

### 2.8 Rigor

For the trustworthiness of the data, the Lincoln and Guba criteria were followed for credibility, transferability, dependability, and confirmability (Ahmed, 2024; Ritter et al., 2023). The researchers used triangulation during the analysis, along with member checking, a complete description of the study, a reflexivity process, and a description of the coding process from the narratives to the final themes.

## 3 Results

We used Walsh’s (2002) proposal to organize the results, considering the following as main categories: family belief system, organizational patterns, and communication processes.

### 3.1 Family belief system process

#### 3.1.1 Making meaning of adversity

While some families needed to find a causal explanation for what was happening to them, others seemed not to need it and to live with the uncertainty. Most participants categorized their child’s illness as an unexpected event; some men reported not knowing the causes and some women reported that there was a genetic predisposition.

*“He has no history of cancer; it was sudden; I have not accepted a cause of that disease yet.” (P5)*

Additionally, many families turned to faith and rituals to make sense of the adversity they were experiencing. Although most participants reported belonging to a religion, others said that they did not engage in specific spiritual practices. Even so, half of the sample mentioned situations of spiritual growth or personal values such as faith and love.

*“Well, we were…we believe in God, and we were very distant before, we did not go to church very much; but now, yes, I have become very attached to God and it seems that God gives us the strength and faith to go on.” (P6)*

*“…I’ve only had one thought…trusting in God that he will be healed.” (P5)*

While half the sample reported having experienced an adverse social or clinical situation before the cancer diagnosis, the other half reported not having experienced anything similar before. Additionally, some participants felt that the process, although difficult at first, was a positive change for their children and did not affect the family dynamics. However, when discussing a sense of adversity, much of the sample expressed the possibility of death and other tragic consequences of the disease, as well as a perception of difficulty.

*“At this time, our concern is that things do not get worse, that is, that we do not get to an extreme point where she does not beat the cancer; that’s our fear.” (P7)*

*“At first, it was tragic, devastating…everything fell apart, work, the stability of being with the family; yes, because mom is at home and I’m here, you know? The entire nuclear family fell apart.” (P3)*

*“‘…I’m very concerned that my son’s treatment is adhered to, because with the problem with the EPS [Colombian Health Promotion Agencies], it can be delayed and with cancer that means a relapse.” (P5)*

#### 3.1.2 Positive outlook

Concerning the parents’ perspective of the disease, we found that only one-third of the men and women were resilient to the process of their child’s disease. In other words, they maintained a positive view, while the rest of the sample did not share that outlook. Notably, some parents found strength in the small details of everyday life, such as their children’s positive reactions, gifts from strangers, or the love expressed within the family.

*“…the strength you draw on in moments when you least expect to have strength, in the strength you see in her at 7 years old, getting what she’s getting, the strength that each of her brothers and sisters puts into understanding it, because at 5 and 3 years old, what can they understand? But they carry on and love, because there is love all around this, there is a lot of love, the love you give her first, the love her siblings give her, the love given by people who sometimes do not even know you, but find out and then send you a message or come to you with a gift, and then you say that’s love, love from people and then the love of God. As I told you, I have never felt so loved by God as I do right now, in this process of living through these six months that we have been going through.” (P1)*

When talking about positive thinking, hope, and optimism, most parents reported that they felt encouraged to handle the situation and to maintain a positive attitude. Additionally, imagining better living conditions and observing the current situation helped some parents in the study overcome the reality of the disease. Religious beliefs also help them maintain a positive outlook on the process of the illness and interpret the suffering caused by this situation as a part of life.

*“…for us, illness has been a situation of growth, of unity, of learning to love, learning to be strong. Suffering, as you know, and as you asked me, I am Catholic, as we are all Catholic in this house, so suffering is something we understand, something that is necessary in life, something that all people have to go through. We all experience it as an illness, and we must know how to bear suffering, live through it with dignity, and come out of it a better person. Otherwise, you have wasted your time suffering, and it would have been better not to have suffered at all (laughter). So, we believe that suffering and pain are essential.” (P1)*

In some cases, these positive views coexisted with negative perspectives. Asked about expressions of defeatism and suffering in the children, only one of the mothers identified with this, while no men did. Although most parents expressed fatalism at the onset of the disease, this then eased or faded away.

*“Well, right now the concern is that things do not get worse, that we do not reach an extreme situation where she does not survive the cancer. That’s our concern.” (P7).*

*“…something very hard. For me it was very difficult, because she has always been very healthy, she never gets sick, and then one day they come and tell you that your child has cancer. It’s very hard.” (P2)*

*“At first it was tragic, devastating.” (P10)*

#### 3.1.3 Transcendence and spirituality

Concerning transcendence and spirituality as elements of belief, as proposed by Walsh, most of the parents in the study reported belonging to a religion, with men identifying as Catholic and women identifying as Catholic or otherwise Christian. These different experiences and encounters with religion are reflected in our sample.

*“…one of the most important thoughts that stays with me is that God is faithful, that God loves us, that God has allowed this illness so that we can be better, that God’s plan is perfect for everyone, not just for X, but for each member of this family.”(P1)*

*“…prayer is something that helps us a lot, from the youngest – well X (the patient’s sister) does not pray – but for Y (the patient’s brother) and everyone older, it’s like strengthening us in prayer.” (P1)*

*“We are Catholics, Mass on Sunday, rosary during the week, frequent confessions, we observe Lent very intensely, with a spiritual direction. This has contributed in every way to the hope that is placed in heaven, the love of the cross, because you have it because Jesus is on the cross, so you see the suffering as that cross that He carried before for you. The support I have from that part of the movement; the consecrated women and the priests have been very much present.” (P1)*

Among the parents who participated in the study, no significant changes were observed in their or future plans or dreams for the family after the diagnosis of the disease. Nor were there any changes in their spiritual experience. In terms of dreams for the family, the mothers – more than the fathers – expressed an urge to raise their child, while both stated that their dreams were to live as a family and bear witness to the experience. The most significant positive lesson for the participants was related to valuing life (mothers), union (fathers), and for both, the strength of the patient and facing difficult moments. Although not widespread, an increase in family unity in the study sample showed an alteration in terms of a change in the meaning of life.

*“…let us say that I changed in a way because I would say that is both negative and positive. Well, it’s negative because the four of us no longer share the same space together; it’s no longer the same environment, so let us say that is the negative thing, that we are isolated; for example, I do not spend time with my other son, my wife spends no time with the girl, or vice versa. the children do not hang out together; they have not seen each other for more than a month, so that’s the negative thing. And on the positive side, it’s taught us how to be more, how to have more personal love for them, love as a union also in the relationship, and how to overcome obstacles that come our way.” (P7)*

### 3.2 Organizational processes within the family

#### 3.2.1 Perception of change and flexibility

In the study, a majority of both the men and women reported a perception of difficulty in adapting to changes, mainly those resulting from patient care, family care, and personal time. However, a noteworthy positive change was that they reported sharing more as a family.

The strategies most frequently used by the participants in this area included communication within the family, staying together, as reflected in the family union, the distribution of burdens, and maintaining routines. In contrast, aspects like finding it difficult to have their own space and personal time and a positive response in general hindered flexibility in the family. Significantly, most of the participants did not employ any strategies to adapt to the changes, which may be related to the reported difficulty in adapting to changes and the difficulty in finding personal space and time. Additionally, for the study participants, reorganization among the family members manifested itself more in the patient’s siblings and parents.

Most of the participants stated that they believed that changes are important. However, there was some ambivalence about whether the changes were perceived as calamitous or difficult, but necessary. Additionally, for most participants, the changes were not perceived as something that would help them grow.

*“Everything changed; our routine was to take her to school, pick her up, do homework, go out on weekends to a park. We do not go out with her anymore. The routine is: go to the hospital, go to the doctor’s appointments, to chemo, take care of her diet, take care of her going out, she cannot run or jump. Everything depends…everything revolves around her.” (P6)*

*“… this has served to strengthen our bond as father and daughter, so let us say that something good came from something bad. But something bad also came, like having to distance myself from my son, since that bond is not as strong. My relationship with my wife, well, I think it’s kind of monotonous; she’s at her job and I’m here, we come and chat for a while, she leaves, I stay or vice versa…” (P7)*

Regarding the displacement of personal activities, only two of the men in the sample identified with this, while none of the women did. In the context of the sample, displacing personal activities became a secondary option due to a fear of loss, and thinking about the work overload involved in caregiving tasks was preferable. Only one of the caregivers responded that they prioritized their own wellbeing, indicating a widespread lack of self-care when coping with the disease. A few of the parents emphasized a negative view of the changes experienced, one-third of the men in the sample as compared to only one of the women.

#### 3.2.2 Connectedness

Here, we found that for some parents the impact of the disease on the family bonds was more difficult, as was the negative impact on the couple’s relationship after their child’s illness. Indeed, several interviewees noted an improvement in family ties.

*“It brought us closer together, like we bonded more, we fight less, we are more united.” (P6)*

*“…here you learn a lot, you learn more about unity, love for your own family, you learn to recognize who is with you through thick and thin, you learn to appreciate more what you do, your everyday life.” (P7)*

However, with regard to repairing and strengthening the family, both fathers and mothers expressed a positive outlook, although family reflection was not evident in the sample. Finally, the parents in the sample perceived that the emotional state of the family depended largely on the caregiver.

#### 3.2.3 Social and economic resources

With the social and economic resources, no significant differences were found between fathers and mothers. In the sample, the extended family was reported as the main source of support, followed by friends and co-workers, with the spouse not being perceived as the first source of support. The interviewees, both men and women, rarely expressed the need to seek additional social support, with only one person in the sample identifying with this strategy. Regarding the belief in the need to strengthen networks, three of the fathers identified with this strategy, in contrast to one of the mothers.

When asked about support from schools, both the fathers and mothers in the study perceived greater support from educational institutions at a psychological and social level, but not when the child’s academic process during treatment was involved.

*“…well, educationally speaking, no, I have not noticed it. They had promised that every eight days they would send us work for us to do with the girl. I enrolled her with the teacher here, so we agreed that she would be the support, the work would come from the school, but the teacher would be the one to help us, let us say, to solve the problems they sent from there to here. But no, they have not sent any activities so far. That’s academically speaking, because personally, they did once collect some things from the children and they sent her some little things, they sent her money, they sent her a letter, which was really nice, also something nice to cheer her up.” (P7)*

*“Not so much, we are currently teaching second grade on our own, because the school has not said anything.” (P1)*

The characteristics of the Colombian healthcare system influenced the narratives about resources. Although regulations stipulate that the system must cover the care required for pediatric cancer, the families were significantly impacted financially by the disease. Both fathers and mothers reported a negative impact on their financial resources, little economic support from the family, and concern about wives continuing to work. As one father explained:

*“…it’s complicated, because if I do not work hard, it’s hard to keep making money, right? So let us say that right now we only get by with my wife’s income, but I’m afraid of losing my job.” (P7).*

Another parent emphasized the fragility of insurance continuity:

*“…it’s worrisome; right now I do not have a job and pretty soon I’ll be out of the health care system and, therefore, my son will be too. This is what mostly makes me sad and worried.” (P9)*

Read through Walsh’s lens, these accounts illustrate how organizational patterns are directly modulated by systemic contingencies (job retention, contributory status, administrative timing), with downstream effects on beliefs (hope vs. uncertainty about sustained treatment) and communication (frequency and quality of interactions with services and support organizations).

### 3.3 Communication processes in the family

#### 3.3.1 Openness to emotional expression

Most of the people interviewed were open to expressing their emotions, conveying positive emotions to a greater extent than negative ones. The study found that the emotion most expressed by the parents was joy, followed by anguish and fear. Women reported feeling emotions such as anxiety, harmony, and restlessness less frequently, while for men the least expressed emotions were nostalgia, peace and calm, emotional volatility, and sadness. However, there was no evidence of relevant differences in the frequency of expressing emotions between men and women.

*“…except anger, they are all valid… you can get frustrated, you can cry, you can be sad; the mother cries first, the father also shows his crying side a lot, because I think he cries more than the mother in certain cases, so in that sense we do have permission to do so.” (P1)*

*“…oh no, forgive me, but this week I was depressed for two days, and then I’ve been happy two days; for example, my mood* var*ies a lot, when she’s bad, it makes me bad, when I see her well, I’m fine…and in the family, I’d say the same occurs.” (P7)*

As for the conditions behind the expression of emotions, both fathers and mothers reported having a reliable family environment in which to emote express emotion, found it easy to ask the family for help, and appreciated the use of humor to defuse difficult moments.

*“Well, I think that the joy, the good humor of X (the patient’s brother) makes us laugh a lot, the peacefulness of Y (the patient’s sister). Yes, like that calm that she gives us because she’s a baby and everything is simpler to her. I think that a good mood in general, a good mood in the house…I think that emotion helps a lot.” (P1)*

The parents in the study did perceive challenges in expressing emotions within the family, with anger and sadness being the emotions that were the most difficult to voice for men. In the same way, the men and women in the sample clearly found it hard to convey their emotions in front of the patient.

*“…I cannot show weakness in front of her, I cannot cry, even if I want to; I have not cried in front of her… my son (the patient’s brother) is very quiet; he does not express much. My wife… it’s been hard for her, logically, because she is with her all the time, so she had some difficult moments at the beginning, as our daughter was there and I try to talk to her and support her, but it’s difficult.” (P4)*

The most common activities that helped the interviewees express their emotions were, in order: playing, talking, going out to shopping centers, going to the movies, and family lunches.

#### 3.3.2 Clarity

With respect to the forms of communication in the family most commonly used by the participants, we primarily found a non-omission of information within the family nucleus, the existence of communication channels between all the members, and various means used to communicate with each other. The results also showed a general affirmative response to the existence of clear and frank communication within the family, as well as a relationship between clear communication and personal support.

*“Being more united…. we had more communication.” (P4)*

*“We did not used to communicate as much as we do now; this has been really important, a great support.” (P5)*

When it came to considering outside help with communication, half the participants stated that they had sought support during their child’s illness from various professionals, such as psychologists, psychiatrists, or physicians. However, most had not considered help from a close third party to facilitate communication within the family.

#### 3.3.3 Collaborative problem solving

The study sample did not reveal any easy ways to find solutions. However, within the collaborative solution strategies of family support, assistance, or care found in the sample, it was evident that dialogue as a strategy was significant, and that it served as a form of support among the family members. Creativity, on the other hand, was not found to be as significant for either mothers or fathers.

*“Speaking and discussing as a family has helped us a lot; this has strengthened us and brought us closer together.” (P1)*

## 4 Discussion

Our study identifies how parents of children with leukemia in Colombia overcame adverse circumstances by using their own resilience repertoires, consistent with the three domains of Walsh’s model (2002): beliefs, organizational patterns, and communication processes. Families moved between narratives of distress and resilience during the course of the illness, reporting greater distress early in the diagnosis and greater resilience as time progressed, an evolution consistent with Huang et al. (2025), who describe an initial disruption phase followed by adjustment and later growth.

Throughout the belief, organizational, and communication processes, we identified transversal elements, such as family-centered orientation and affective bonds, and both optimistic and pessimistic thoughts. Crucially, in Colombian healthcare – where pediatric oncology care is accessed, coordinated, and sustained through regulated pathways and service arrangements – these contextual conditions operated through the three resilience domains rather than around them. They shaped belief systems (e.g., hope vs. uncertainty regarding uninterrupted treatment and access), configured organizational patterns (e.g., role redistribution to safeguard income, preserve insurance continuity, and navigate administrative procedures), and conditioned communication processes (e.g., regular coordination with rotating providers and institutions). In this context-embedded dynamic, transcendence/spirituality and available financial resources functioned as cross-cutting supports for meaning-making and organization, while a positive attitude toward the trajectory of the illness and the child’s future – often expressed in terms of long-term family planning – further strengthened resilience.

Within belief systems, spirituality emerged prominently as a source of hope and emotional management. Practices such as family prayer, participation in religious communities, and support from church members aligned with findings by Nicholas et al. (2017), Matos and Mercerón (2017), and Wiener et al. (2016), who describe spirituality as guiding decision-making, providing emotional grounding, and contributing to meaning-making and perseverance. For the participants in our sample, these practices provided similar benefits. Along the same line, some families understood illness as an opportunity to value life, unity, and personal growth. While this contrasts with studies whose appraisals were more negative (Matos and Mercerón, 2017; Peterson et al., 2014), our participants at times expressed hopeful or future-oriented thinking.

In our sample, the positive outlook described in the earlier literature was not a dominant, stable pattern. In contrast to studies that report that a positive appraisal of the illness is strongly associated with family resilience (e.g., Hinton et al., 2022; Qiu et al., 2024), only one-third of the parents in our study consistently maintained a positive view of their child’s illness. While some parents found strength in small everyday moments – their child’s emotional responses, expressions of affection within the family, or gestures from strangers – most did not maintain a positive perspective throughout the process. For many families, early reactions were characterized by fatalism, shock, and distress which, while they gradually diminished, did not fully transform into sustained optimism. This coexistence of hopeful elements and negative appraisals in our participants’ experiences over time indicate that, although a positive outlook can foster resilience, it is not a universal or stable feature. Instead, positive and negative interpretations fluctuated over time, suggesting that meaning-making around the illness was dynamic and context-dependent, rather than uniformly resilient.

Regarding organizational patterns, our findings align with Walsh (2002), who identifies flexibility, emotional connection, and access to resources as key. The families in our study reorganized roles, schedules, and responsibilities, consistent with the research done by Rubio Morell et al. (2014), Grau and Espada (2012), and Arruda-Colli et al. (2018). However, the negotiation difficulties, gendered power dynamics, and limited flexibility described by Matos and Mercerón (2017) – especially in traditionally gender-structured families – were also evident. Employment adjustments, as noted by Brody and Simmons (2007), were common as well. While role shifts enabled families to preserve income, coverage, and treatment continuity, they also concentrated caregiving burdens on mothers, while men continued in their jobs, albeit with their routines disrupted. Although this gendered asymmetry seems to be functionally adaptive in the short term, it may lock in gender roles and intensify inequalities if sustained, indicating a resilience pathway that is simultaneously effective and unequal.

In terms of economic and social resources, financial concerns were also prominent, echoing Athié and Gallegos (2009), who describe socioeconomic status as shaping resilience and future outlooks. While the families acknowledged some institutional support, the bureaucratic barriers in the Colombian healthcare system created uncertainty and added financial strain, contributing to a sense of vulnerability. Similarly, inconsistent and short-term professional psychosocial support – provided by different specialists each time – limited the perceived usefulness of these services. On the contrary, extended family, friends, and co-workers constituted key social resources, in line with the findings by Matos and Mercerón (2017), Van Schoors et al. (2015), and Doumit and Khoury (2017). Spousal support varied, but when couples effectively mobilized resources, their relationships became stronger (Arruda-Colli et al., 2018).

The wellbeing of the parents frequently deteriorated, mirroring Wiener et al. (2016): they postponed personal activities, adopted unhealthy habits, and experienced stress-based physical decline. Unlike Ponce Espino and Torrecillas Martín (2014), we found no marked emotional differences between men and women in our sample. The parents’ reports of postponed self-care, deteriorating routines, and stress-linked health complaints indicate that the primary caregiver’s wellbeing is a fragile node in the family system. Moreover, while it enables them to adapt to daily routines, it is chronically under-resourced. With regard to Walsh’s model, this undermines organizational capacity over time and increases the communication workload required to keep care coordinated, illustrating how resilience is at times maintained at the cost of caregiver depletion. Furthermore, if these adaptive patterns persist, families can face cumulative costs: caregiver exhaustion (usually affecting women), reduced flexibility to rebalance roles, and a narrowing of organizational options in the midst of ongoing financial and administrative pressures. Conceptually, this results in resilience trajectories that are stable yet brittle, effective in maintaining treatment continuity, but vulnerable to shocks and setbacks.

Finally, the communication processes reflected Walsh’s (2004) emphasis on emotional expression, clarity, and collaborative problem solving. Parents often refrained from expressing distress in front of their children, but reported intense internal emotional struggles, echoing Brody and Simmons (2007). As shown by Rosenberg et al. (2013) and Doumit and Khoury (2017), open communication within the family and with clinical teams reinforces resilience. Our findings also show strengthened family bonds, consistent with Huerta and Rivera (2017). Difficulties expressing negative emotions, however, particularly among men, limited conflict resolution, reflecting the patterns described by Matos and Mercerón (2017). In this respect, dialogue emerged as a significant supportive strategy in our sample, while creativity played a lesser role (Arruda-Colli et al., 2018). Consistent with Sidhu et al. (2005), open communication facilitated a more positive outlook about the illness.

Taken together, our data suggest that resilient reorganization and vulnerability frequently co-occur rather than develop sequentially. Families mobilized meaning-making, role redistribution, and communication to meet day-to-day demands, while simultaneously grappling with uncertainty, fatigue, and financial strain. This coexistence indicates that resilience is better understood as an adaptive process under constraint, not as the absence of vulnerability. Our results reflect broader patterns of adaptation described by Van Schoors et al. (2015) and Rosenberg et al. (2013), who report phases of chaotic functioning followed by eventual stabilization. The parents in our study perceived the caregiver’s emotional state as central to family wellbeing, consistent with findings by Matos and Mercerón (2017).

Overall, our study substantially contributes to the literature by providing the first qualitative, context-sensitive analysis of how Colombian families of children with leukemia draw on beliefs, organizational patterns, and communication processes to sustain resilience over the first year post-diagnosis. Our findings have several important implications. First, they extend the application of Walsh’s model to a Latin American context, showing that spirituality, extended family involvement, and adaptive role redistribution operate as culturally embedded resilience mechanisms during the first post-diagnosis year. Second, the results highlight structural vulnerabilities – financial strain, inconsistent psychosocial support, and barriers in the healthcare system – that directly shape families’ organizational capacity and need to be considered in family-centered interventions. Third, the study underscores the importance of supporting the primary caregiver’s emotional wellbeing, given its central regulatory function in the family environment and in adaptive functioning. Collectively, these findings suggest specific and context-appropriate targets for strengthening resilience processes in families dealing with childhood leukemia in Colombia.

### 4.1 Limitations

Our results should be interpreted in light of the study’s methodological constraints. First, the small sample size and the limited diversity of the participating families restricted the breadth of the perspectives represented and could reduce the transferability of the findings. Furthermore, all the participants were recruited from the same non-profit organization, which may have constrained the findings by yielding a limited perspective. Future research should broaden the recruitment strategy to include participants from other associations, as well as from public and/or private institutions. Second, the sampling strategy introduces a potential selection bias, as the families who agreed to participate may differ systematically from those who did not, particularly in terms of coping style, availability, or willingness to discuss their experiences. Third, although gender was considered in the interpretation of the narratives, it was not a central analytical axis, which limits the depth of the interpretation of gender-related differences in resilience processes. Additionally, the inclusion criteria focused only on families who remained in treatment during the first year, meaning that parents who lost their children during this period were not included; future studies should analyze these experiences to identify resilience factors that may differ in bereavement. Finally, further qualitative work is needed to compare these findings with families coping with other childhood cancer diagnoses, different prognoses, or more complex comorbidities, as well as with families who experience a more favorable recovery trajectory during the first year post-diagnosis.

## 5 Conclusion

The results of this study identified the resilience factors present and absent in a Colombian sample of parents of children with leukemia. Although it was possible to apply Walsh’s proposal to the selected Latin American sample, the elements must be interpreted taking the historical, cultural, and chronological context into consideration. Identifying the factors that favor or hinder an adequate resilience process for parents provides information that can be used to craft appropriate and timely interventions aimed at improving mental health, accompanying decision-making, strengthening family relationships through communication, and improving the relationship between parents and their child, improving the family’s quality of life and promoting spaces for wellbeing (Park et al., 2022).

Finally, this study sought to determine the particular elements that bring different professions into dialogue with each other in order to promote interdisciplinary therapeutic proposals. Based on data from the health and social sciences, these proposals make it possible to develop holistic, humanized interventions that respond to the needs of these families -not only physical and emotional, but also social and spiritual- to encourage family involvement, personalized planning, and flexibility and adaptability to respond to the changing needs of the family at the different stages of their child’s cancer process.

## Data Availability

The original contributions presented in the study are included in the article/Supplementary material, further inquiries can be directed to the corresponding author.
