# Correction: Real-world experience with family beliefs and organizational and communication management in Colombian parents of children with leukemia: a qualitative study

**DOI:** 10.3389/fpsyg.2026.1931922

**Published:** 2026-07-23

**Authors:** Luz Helena Buitrago-León, Yolanda Pastor, Domingo Palacios-Ceña

**Affiliations:** 1 International Doctoral School, Rey Juan Carlos University, Móstoles, Spain; 2 Research Group in Health, Sport and Clinical Psychology, Faculty of Psychology, Universidad El Bosque, Bogotá, Colombia; 3 Department of Psychology, Research Group on Health and Psychosocial Well-Being in Adolescence and Youth (WELL-YOUTH), Universidad Rey Juan Carlos, Alcorcón, Spain; 4 Department of Physical Therapy, Occupational Therapy, Physical Medicine and Rehabilitation, Research Group of Humanities and Qualitative Research in Health Science (Hum&QRinHS), Universidad Rey Juan Carlos, Alcorcón, Spain

**Keywords:** childhood cancer, family beliefs, family communication, family resilience, leukemia, organizational processes

Affiliation “International Doctoral School, Rey Juan Carlos University, Móstoles, Spain” was omitted from the paper. This affiliation has now been added for author Luz Helena Buitrago-León.

The original version of this article has been updated.

