# Supplementary material for: Real-world experience with family beliefs and organizational and communication management in Colombian parents of children with leukemia: a qualitative study
Source: Front Psychol. 2026 Apr 1;17:1783044. doi: 10.3389/fpsyg.2026.1783044 (PMC13079619; doi:10.3389/fpsyg.2026.1783044)
Supplement: Supplementary file 1 [file Data_Sheet_1.pdf]

Supplementary Material

1 Supplementary section 1. Context

The care of children with cancer in Colombia is regulated by several laws and regulations that seek to guarantee comprehensive and priority care for this population. Among the related laws regulating healthcare in Colombia are: 1) Law 1388 of 2010 (https://www.minsalud.gov.co/sites/rid/Lists/BibliotecaDigital/RIDE/INEC/IGUB/cancer-infantil-Ley-1388-2010.pdf), which establishes the right to life for children with cancer and regulates comprehensive and priority care for this population, regardless of the family's insurance status (contributory or subsidised); 2) Law 2026 of 2020 (https://www.funcionpublica.gov.co/eva/gestornormativo/norma.php?i=159507), which expands Law 1388 of 2010 and establishes specific measures to guarantee the provision of paediatric oncology services; and 3) Resolution 994 of 2022 (https://minsalud.gov.co/Normatividad_Nuevo/Resoluci%C3%B3n%20No.%20994%20de%202022.pdf), which establishes the mechanism for accessing and using data and reports on ruinous and catastrophic high-cost diseases. Care is delivered through accredited oncology units and specialized hospitals, offering multidisciplinary services including chemotherapy, radiotherapy, psychosocial support, and nutritional assistance.

According to data from the Pan American Health Organisation (2021), approximately 2,211 cases of cancer are diagnosed annually in children under 18, and in Bogotá, the mortality rate was 6.5 per 100,000 children. Suárez Mattos et al. (2018) reported that although survival rates for acute leukaemia in children improved from 41% (1990–95) to 54% (2005–09), they still lag behind countries with greater resources. These authors denounce the fact that comprehensive healthcare is not available in all cases and areas of the country, and that the system needs improvement. For this reason, non-profit organisations often accompany families throughout the disease process, such as the Colombian Leukaemia and Lymphoma Foundation (Funcolombiana). This foundation aims to support the quest to eradicate cancer, with a focus on research, diagnosis and treatment of blood cancers. It has a childhood cancer programme that offers support to families throughout the disease process, in collaboration with two clinics in Bogotá. They also offer telephone support to families in other parts of the country.

References:

Pan American Health Organization. (2021, September 16). Childhood cancer country profile: Colombia [Infographic]. OPS/OMS. Available at https://www.paho.org/en/documents/infographic-childhood-cancer-country-profile-colombia

Suárez Mattos, A., Aguilera, J., Salguero, E. A., & Wiesner, C. (2018). Pediatric oncology services in Colombia. Colombia Médica, 49(1), 97–101. https://doi.org/10.25100/cm.v49i1.3377

Supplementary Material should be uploaded separately on submission. Please include any supplementary data, figures and/or tables.

Supplementary material is not typeset so please ensure that all information is clearly presented, the appropriate caption is included in the file and not in the manuscript, and that the style conforms to the rest of the article.

2 Supplementary section 2.

Question guide. Available at: https://data.mendeley.com/datasets/ppnfbmz5rg/1/files/deec36d5-54d9-408b-b28a-ed392cc21c26

3 Supplementary section 3.

Conceptual mapping. Available at: https://data.mendeley.com/datasets/ppnfbmz5rg/2
